# The *ACTN3* R577X Nonsense Allele Is Underrepresented in Professional Volleyball Players and Associated with an Increased Risk of Muscle Injury in Female Players

**DOI:** 10.3390/genes16091076

**Published:** 2025-09-13

**Authors:** Mesut Cerit, Selin Yıldırım Tuncer, Muhammed Mustafa Piri, Murat Anılır, George John, Ekaterina A. Semenova, Andrey K. Larin, Edward V. Generozov, Ildus I. Ahmetov, Korkut Ulucan, Attila Szabo

**Affiliations:** 1 Department of Exercise and Sports Science, Lokman Hekim University, 06510 Ankara, Türkiye; mesut.cerit@lokmanhekim.edu.tr (M.C.); 221760002@lhu.edu.tr (M.M.P.); 2 Department of Sports Management, Lokman Hekim University, 06510 Ankara, Türkiye; 3 Transform Specialist Medical Centre, Dubai 119190, United Arab Emirates; drjohnsportsfit@hotmail.com; 4 Department of Molecular Biology and Genetics, Lopukhin Federal Research and Clinical Center of Physical-Chemical Medicine of Federal Medical Biological Agency, 119435 Moscow, Russiagenerozov@gmail.com (E.V.G.);; 5 Research Institute of Physical Culture and Sport, Volga Region State University of Physical Culture, Sport and Tourism, 420138 Kazan, Russia; 6 Laboratory of Genetics of Aging and Longevity, Kazan State Medical University, 420111 Kazan, Russia; 7 Research Institute for Sport and Exercise Sciences, Liverpool John Moores University, Liverpool L3 5AF, UK; 8 Faculty of Dentistry, Basic Medical Sciences, Department of Basic Health Sciences, Marmara University, 34722 Istanbul, Türkiye; 9 Faculty of Health and Sport Sciences, Széchenyi István University, H-9026 Győr, Hungary

**Keywords:** DNA, polymorphism, genotype, volleyball, talent ID, sport injuries

## Abstract

Background: Muscle injuries pose a significant challenge in sports, leading to decreased performance and shortened career longevity. Individuals homozygous for the nonsense X allele of the *ACTN3* rs1815739 (R577X) polymorphism, characterized by a complete absence of α-actinin-3, have been associated with reduced power performance and may have an increased injury risk. This study aimed to investigate the association between the *ACTN3* R577X polymorphism and both volleyball player status and the risk of non-contact musculoskeletal injuries in female volleyball players. Methods: The study included 5382 Turkish and Russian subjects of European descent (187 professional volleyball players and 5195 controls), of whom 50 female players provided injury data. Sport-related injury information was obtained from medical records maintained by team physicians and physiotherapists. Results: A pooled analysis of the two cohorts demonstrated that the frequency of the *ACTN3* X allele was significantly lower in volleyball players than in controls, with an odds ratio of 0.763 (95% CI: 0.61–0.95, *p* = 0.02). In the pre-specified recessive contrast (XX vs. RR + RX) among 50 players, exact methods indicated higher injury odds for the XX genotype (OR = 7.87, 95% CI: 0.94–374.58; *p* = 0.0366), which was classified as borderline/exploratory. Penalized (Firth) regression produced estimates of a similar magnitude after adjustment for age and playing position (adjusted OR = 5.92, 95% CI: 1.12–60.98), although confidence intervals remained wide. Conclusions: The *ACTN3* X allele is underrepresented in professional volleyball players, and it is associated with an increased risk of muscle injury in female players.

## 1. Introduction

Muscle injuries are common, especially among professional athletes, and can greatly affect performance and the length of sports careers. Despite preventive measures, the rate of muscular injuries remains high [1]. According to Solé et al. [2], 75.2% of injuries in professional female volleyball players happen during training, 20.3% during competition, and 4.5% during conditioning drills. Extended high-intensity training may increase injury risk and negatively impact athletes’ physical, mental, and social health [3]. This highlights the importance of studying the role of genetic and other intrinsic factors in injury risk and recovery.

Research on athletes and non-athletic populations using whole genome sequencing, micro-array genotyping, whole exome sequencing, and single genetic variant genotyping has provided new insights into the genetic factors associated with athletic performance and injuries [4,5,6,7,8,9]. Studies on single-nucleotide polymorphisms (SNPs) suggest that specific genetic variants may influence the likelihood, severity, and recovery time of muscle damage. Understanding these genetic contributions is not only of scientific interest but also of practical relevance: identifying genetic predispositions may allow for early recognition of athletes at higher risk, enabling targeted interventions, optimized training loads, and personalized recovery strategies.

Notably, the R577X polymorphism (rs1815739) in the α-actinin-3 (*ACTN3*) gene has been shown to affect fast-twitch muscle fiber composition and athletic performance [10,11]. This polymorphism results from a cytosine-to-thymine (C>T) substitution at nucleotide position 1747 in exon 16, leading to the replacement of an arginine residue at codon 577 with a premature stop codon. The R577X variant has been associated with muscle strength, speed, and injury susceptibility [8,12,13].

Individuals with the XX (TT) genotype exhibit a functional deficiency of α-actinin-3, which leads to increased transcript and protein levels of Z-disk proteins such as myotilin, desmin, and γ-lamin. This alteration increases susceptibility to musculoskeletal damage [14,15]. Studies indicate that athletes with the XX genotype are more prone to non-contact muscle injuries compared to those with RR and RX genotypes, which produce functional α-actinin-3 in fast-twitch muscle fibers [8,16,17].

The RR genotype of the *ACTN3* gene has been associated with increased muscle stiffness, which may serve as a protective mechanism against injury [11]. In muscle physiology, α-actinin-3 plays a crucial role in actin filament organization and Z-disk integrity. The ACTN3 protein is exclusively expressed in fast-twitch (type II) muscle fibers, which are essential for explosive and high-intensity activities such as sprinting, weightlifting, short-distance swimming, soccer, volleyball, and basketball [13,18,19]. Athletes with the RR and RX genotypes exhibit superior fast-twitch muscle fiber function compared to those with the XX genotype, who rely more on slow-twitch muscle fibers. The presence of α-actinin-3 is critical for maximizing the strength of type II muscle fibers [20].

Therefore, clarifying the association between *ACTN3* and injury susceptibility is clinically meaningful, as it may eventually support individualized training prescriptions, monitoring strategies, and injury-prevention programs in professional athletes. This study aimed to investigate the association between the *ACTN3* R577X polymorphism and both volleyball player status and the risk of non-contact musculoskeletal injuries in volleyball players.

## 2. Materials and Methods

### 2.1. Ethics Statement

The study was conducted following the Declaration of Helsinki, and ethical approval was obtained from the Lokman Hekim University Non-Interventional Clinical Research Ethics Committee (reference 2025-110; approval date: 28 April 2025) and the Ethics Committee of the Federal Research and Clinical Center of Physical–Chemical Medicine of the Federal Medical and Biological Agency of Russia (reference 2017/04; approval date: 4 July 2017). Informed consent was obtained from all participants.

### 2.2. Participants

The study included 5382 Turkish and Russian subjects of European descent, comprising 187 professional volleyball players and 5195 controls.

Of the 187 volleyball players, 50 were elite Turkish female players (mean age: 26.2 (4.7) years), representing five clubs in the professional women’s volleyball league and providing injury data. Eligibility criteria required Turkish volleyball players to have participated in the professional volleyball league for a minimum of 4 years and a maximum of 12 years. Only players who had sustained significant muscle tissue injuries and resumed their playing careers after 3–4 months of treatment were included. All other forms of injury, including fractures, sprains, contusions, and surgeries, were excluded. During the volleyball season, these players participated in one official match per week and engaged in six training sessions lasting approximately 45–75 min, focusing on technique, tactics, and conditioning.

All injuries were corroborated by official medical records, and only recorded cases were incorporated into the study. Diagnoses were determined by team physicians using standardized clinical examinations and, when required, corroborated by imaging techniques such as magnetic resonance imaging (MRI) or ultrasound. The severity of the injury was categorized based on the length of time away from training and competition. Injuries that caused a delayed return to sport of three to four months or more were classified as severe. Minor muscular strains are frequently underrepresented in medical documentation and may be recalled inconsistently by athletes, especially for the severity of the injury [21]. Consequently, limiting the analysis to severe injuries was considered essential to enhance methodological consistency and mitigate reporting bias.

For comparison, *ACTN3* R577X genotype data from 557 healthy Turkish controls were obtained from the Turkey National Genome and Bioinformatics Project [22]. These controls had no history of elite sport participation. Detailed information on recreational physical activity or training load was not available; however, the cohort represents the general population and met strict inclusion criteria to exclude individuals with chronic or hereditary diseases. All Turkish athletes and controls were of Caucasian ancestry.

The Russian athletic group included 137 professional volleyball players (50 females, mean age (SD) 24.8 (4.7) years; 87 males, mean age (SD) 27.7 (5.6) years), of whom 25 were classified as highly elite (prize winners in international competitions, including the Olympic Games and World Championships), 33 were elite (prize winners in national competitions), and 79 were sub-elite (non-prize winners in national competitions). Muscle injury data were not available for the Russian athletes, as this cohort was included solely to compare *ACTN3* allelic frequencies between athletes and controls. The Russian control group consisted of 4638 individuals, including healthy donors and patients with multifactorial conditions, as previously described by Barbitoff et al. [23]. Detailed data on physical activity or sports participation were not available for these controls. All Russian athletes and controls were of Caucasian origin.

### 2.3. Genotyping

Oral swab samples from Turkish volleyball players were collected for DNA analysis by appointment before training sessions during the season. DNA extraction was performed on the collected samples, and the *ACTN3* rs1815739 polymorphism was analyzed using Real-Time PCR. All genetic analyses were conducted in collaboration with Zip Prime Biotechnology. Oral swab samples were obtained using the Buccal Swab Collection Kit and stored at +4 °C under appropriate laboratory conditions. Genomic DNA was extracted using the ZipPrime Epithelial Cell DNA Extraction Kit. SNP analysis of the rs1815739 polymorphism was carried out using the ZipPrime™ Lifestyle Real-Time PCR Kit (Athletic Performance Module, Ankara, Turkey), with genomic DNA obtained from the athletes. Probes labeled with HEX were used for SNP detection. Genotyping was performed using the SLAN 96-P Real-Time PCR system, employing Fluorescence Melting Curve Analysis (FMCA) for precise allele discrimination.

For the Russian athletes, DNA was extracted from leukocytes isolated from 4 mL of venous blood. The extraction and purification processes were carried out using a commercial kit following the manufacturer’s protocol (Technoclon, Moscow, Russia). Genotyping of the *ACTN3* R577X polymorphism was performed using microarray technology (Illumina, San Diego, CA, USA) with HumanOmni1-Quad and HumanOmniExpress BeadChips (Illumina), as previously described [24]. Genotype data for the Russian control group were obtained through whole-exome sequencing, as previously described [23], with the reported genotype data available in the RUSeq database [25].

### 2.4. Statistical Analysis

Descriptive statistics for categorical variables are reported as counts and percentages (*n*, %); continuous variables are summarized as mean (SD) and median (min–max). Normality was assessed using the Kolmogorov–Smirnov test. Because several continuous variables deviated from normality, group comparisons used non-parametric tests: Mann–Whitney U for two groups and Kruskal–Wallis H for more than two groups, with Bonferroni-adjusted pairwise comparisons where applicable. Two-sided α was set at 0.05. Given sparse cells in the genotype–injury table, categorical associations were evaluated using two-sided Fisher’s exact tests. A pre-specified recessive contrast (XX vs. RR + RX) was summarized by the exact conditional odds ratio with an exact 95% confidence interval. To address small-sample bias and allow covariate adjustment, we additionally fitted penalized (Firth) logistic regression models with injury status as the outcome and genotype as the exposure. The primary adjusted model included age and playing position a priori; a sensitivity model further included height and body mass. Penalized profile-likelihood 95% CIs and likelihood-ratio *p*-values are reported. For the meta-analysis of allele frequencies, Cochrane Review Manager (RevMan, v5.3, London, UK) was used. Random-effects models were applied; odds ratios (ORs) with 95% CIs were estimated using the Mantel–Haenszel method, and heterogeneity was assessed with the I^2^ statistic. Statistical analyses for non-parametric tests were performed in IBM SPSS Statistics for Windows (v25.0, IBM Corp., Armonk, NY, USA). Exact tests and penalized logistic regression were conducted in R (R Foundation for Statistical Computing, Vienna, Austria) using the stats, “epitools”, and “logistf” packages.

## 3. Results

The characteristics of the Turkish female volleyball players included in the muscle injury study are presented in Table 1.

**Figure 1:**
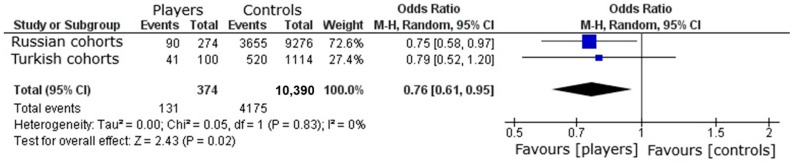
Meta-analysis of association studies examining the *ACTN3* gene and volleyball player status. The under-representation of the *ACTN3* X allele is evident in volleyball players. The purple squares indicate the proportion of the X allele among volleyball players in each individual study, while the black diamond represents the pooled proportion of the X allele across all volleyball players, along with its 95% confidence interval (CI).

The *ACTN3* genotype distribution was consistent with Hardy–Weinberg equilibrium in both Turkish (*p* = 0.3511, χ^2^ = 0.8695) and Russian (*p* = 0.7623, χ^2^ = 0.0915) athletes. The frequency of the *ACTN3* X allele was significantly lower in Russian volleyball players compared to controls (*p* = 0.0285), but no significant difference was observed in Turkish volleyball players (*p* = 0.2752) (Table 2). To jointly analyze the data from both cohorts while accounting for potential population differences, we performed a combined logistic regression with allele status (X vs. R) as the outcome and athlete status (player vs. control) and country (Russia vs. Turkey) as predictor variables. This analysis confirmed a significant under-representation of the X allele in volleyball players after adjusting for country (Odds Ratio [OR] = 0.76, 95% CI: 0.61–0.95, *p* = 0.016). The model also identified a significant difference in the baseline X allele frequency between the Russian and Turkish populations (OR = 0.74, 95% CI: 0.67–0.81, *p* < 0.001). The interaction between athlete status and country was not significant (*p* = 0.83), indicating that the negative association between the X allele and athlete status was consistent across both cohorts. This finding of negligible heterogeneity (I^2^ = 0% and Cochran’s Q = 0.05, df = 1, *p* = 0.83) is supported by a meta-analysis of the two cohorts, which demonstrated that the frequency of the *ACTN3* X allele was significantly lower in volleyball players than in controls, with an odds ratio of 0.763 (95% CI: 0.61–0.95, *p* = 0.02) (Figure 1).

Muscle injuries were reported in 60% (30/50) of Turkish female volleyball players. By genotype (RR/RX/XX), the injury proportions were 57.9% (11/19), 47.6% (10/21), and 90.0% (9/10), respectively, with the lowest incidence in RX and the highest in XX (Table 3). For the overall 3 × 2 table, the two-sided Fisher’s exact test was borderline (*p* = 0.0617). In the pre-specified recessive contrast (XX vs. RR + RX) using exact methods, the odds of injury were higher in XX carriers (exact OR = 7.87, 95% exact CI = 0.94–374.58; *p* = 0.0366); because the exact CI includes 1.00, this result should be interpreted as borderline/exploratory. Small-sample penalized (Firth) logistic regression showed a similar association: unadjusted OR = 5.74 (95% profile-likelihood CI 1.16–57.09; LR *p* = 0.031), adjusted for age and playing position OR = 5.92 (95% CI 1.12–60.98), and further adjusted for height and body mass OR = 5.22 (95% CI 1.02–52.00).

Table 4 indicates that age (*p* = 0.691), height (*p* = 0.834), and body mass (*p* = 0.957) did not significantly differ among genotype groups. However, significant differences in height (*p* < 0.001) and body mass (*p* < 0.001) were observed based on playing position. Players in the libero position were significantly shorter than those in the setter (*p* < 0.001, Bonferroni test) and spiker (*p* < 0.001, Bonferroni test) positions. Additionally, players in the libero position were significantly lighter than those in the middle player (*p* = 0.001, Bonferroni test) and spiker (*p* < 0.001, Bonferroni test) positions. Age, height, body mass, and playing position did not significantly affect muscle injury status.

Multivariable results are summarized in Table 5. In penalized (Firth) logistic regression with injury (yes/no) as the outcome and genotype (XX vs. RR + RX) as the exposure, the unadjusted model indicated higher odds of injury in XX carriers (OR 5.74, 95% profile-likelihood CI 1.16–57.09; model LR *p* = 0.031). After adjustment for age and playing position, the association was similar in magnitude (OR 5.92, 95% CI 1.12–60.98; LR *p* = 0.092). Further adjustment for height and body mass yielded a comparable estimate (OR 5.22, 95% CI 1.02–52.00; LR *p* = 0.314). The wide confidence intervals and the increase in model LR *p*-values with additional covariates reflect limited statistical power; accordingly, the XX–injury association should be interpreted as preliminary/exploratory, despite its consistent direction across models.

## 4. Discussion

Muscle injuries, particularly those resulting from non-contact mechanisms, are prevalent in professional sports such as tennis, volleyball, and track and field, where competitions occur more frequently than in other sports disciplines [1,26,27]. Genetic research indicates that the musculoskeletal system significantly influences soft tissue injuries, accounting for nearly 50% of their incidence [4]. Genetic variations may contribute to differences in injury occurrence, severity, and recovery duration in elite sports [28,29]. From a practical standpoint, such insights may be used to stratify athletes by genetic risk and to develop tailored preventive measures, such as individualized strength conditioning, load management, and recovery protocols.

Research on the *ACTN3* R577X polymorphism in athletes across various sports has shown that the majority of participants exhibit the RR and RX genotypes, with the XX genotype being less common [30,31,32,33,34]. A meta-analysis by El Ouali et al. [35] demonstrated that the distribution of *ACTN3* R577X genotypes follows a linear trend (RX > RR > XX) in sports requiring explosive power. In our study, the genotype distributions among Turkish (RX: 42.0%, RR: 38.0%, and XX: 20.0%) and Russian volleyball players (RX: 44.5%, RR: 45.3%, and XX: 10.2%) followed a similar pattern, with a predominance of the R allele and an under-representation of the X allele in athletes compared with controls (random-effects meta-analysis, OR 0.76, 95% CI 0.61–0.95). This allele-frequency finding concerns athlete vs. control status and should not be conflated with injury risk within athletes. Our findings align with previous research indicating that athletes engaged in sports requiring power and strength tend to possess favorable *ACTN3* genotypes [36].

The higher prevalence of the *ACTN3* R allele among athletes, particularly in sports requiring explosive power and sprint performance, can be explained by its biological role in fast-twitch muscle fibers. The R allele encodes functional α-actinin-3, which is critical for Z-disk stability and force transmission in type II fibers, enhancing sprinting, jumping, and rapid muscle contraction capabilities [10,11,12,15,18]. α-Actinin-3 contributes to mechanical resilience during eccentric contractions by maintaining sarcomere integrity and stabilizing actin filament attachment at the Z-disk [16,30]. In contrast, the XX genotype results in α-actinin-3 deficiency, leading to compensatory upregulation of other Z-disk proteins, altered filament tension, and reduced resistance to structural damage, which may partly explain the higher susceptibility of XX carriers to non-contact injuries [10,14,15]. From an evolutionary perspective, individuals carrying the R allele may have been better adapted to high-intensity physical activity, conferring a selective advantage in activities requiring strength and power [15,20]. In contrast, the XX genotype, which results in α-actinin-3 deficiency, favors endurance-oriented slow-twitch fiber characteristics, which may be less advantageous in power-dominant sports [10]. These biological and evolutionary mechanisms help explain the over-representation of the R allele in elite athletes relative to the general population.

Genetic variation influences both the early phase of post-exercise injury and the subsequent inflammatory phase. Research indicates that *ACTN3* significantly impacts the initial damage phase, with XX genotypes experiencing heightened muscle damage following eccentric loads [16,30], whereas RR genotypes have been associated with lower injury risk [14,37,38]. Our injury analysis (Table 3 and Table 5) showed higher proportions of injury in XX carriers (90%) than in RR/RX; however, exact and penalized small-sample methods indicated borderline/exploratory evidence with wide confidence intervals, reflecting limited precision. Studies with similar findings suggest that individuals with the XX genotype are particularly susceptible to non-contact musculoskeletal injuries [39,40,41].

This susceptibility may be partially explained by hormonal mechanisms. The *ACTN3* R allele has been associated with higher circulating testosterone levels in both males and females [42], which are considered beneficial for the body’s reparative response [43]. Supporting this, recent evidence demonstrates that α-actinin-3 deficiency reduces androgen receptor (AR) expression in skeletal muscle of male and female mice and humans, and blunts the muscle growth response to dihydrotestosterone in female mice at puberty onset [44]. These structural and hormonal mechanisms together provide a solid physiological basis for the protective effect of the R allele against muscle injury.

Our data are consistent with a possible lower injury propensity in RR/RX compared with XX, but estimates are imprecise and should be interpreted as preliminary. In contrast, individuals with the XX genotype exhibit endurance-oriented musculature, characterized by reduced muscular contraction strength. Petr et al. [45] proposed that individuals with the XX genotype may demonstrate diminished isokinetic strength in their quadriceps and hamstring muscles, whereas RX heterozygotes may have a lower risk of injury due to their balanced composition of fast-twitch fibers and endurance characteristics.

One study reported a higher incidence of injuries in runners with the *ACTN3* RR genotype compared to those with the RX and XX genotypes [46]. However, other research has indicated that athletes with the XX genotype experience a higher frequency of non-contact injuries during the season [34] and greater muscle damage following exercise [30]. These inconsistencies across studies may result from differences in sport-specific demands, injury mechanisms, training regimens, and population characteristics. For example, the elevated injury incidence in RR runners may reflect unique biomechanical stresses or repetitive loading patterns inherent to running that are not present in volleyball, whereas our cohort consisted of team-sport athletes with different movement profiles. Moreover, the X allele has been associated with an increased risk of ankle injuries [46,47,48] and a higher overall susceptibility to sports-related injuries [40]. These observations highlight the need for adequately powered, sport-specific studies and underscore the necessity of cautious interpretation when assessing genotype–injury associations.

Thus, within our small volleyball cohort, RR/RX showed lower injury proportions than XX, but this association remains exploratory due to wide CIs and potential confounding. Caution is warranted, and the rs1815739 polymorphism may best be interpreted as a potential risk factor rather than a definitive predictor of individual injury. Further research across diverse training levels and sports disciplines is essential to refine the accuracy of these observations while considering genetic predispositions [49]. Nevertheless, identifying genetic predispositions can help generate risk profiles that may inform the design of personalized training regimens and preventive interventions in athletes. Such applications reflect the long-term potential value of genetic testing in sports medicine, even if immediate clinical translation is premature.

This study has several limitations, including a relatively small sample size of volleyball players with muscle injuries, variations in training regimens and experience levels across teams, limited interaction time with participants, differences in dietary programs, and the examination of only a single genetic variant. Other genetic variants, including *ACE* I/D, *COL5A1* rs12722, *CCL2* rs2857656, *IGF2* rs3213221, *SOX15* rs4227, *TNC* rs2104772, *MMP3* rs679620, *ELN* rs2289360, *ESR1* rs2234693, *COL22A1* rs11784270, *COL22A1* rs657795834, *ESR1* rs223469332, *COL5A1* rs16399, *DCN* rs516115, *HIF1A* rs11549465, *MMP1* rs1799750, *MMP12* rs2276109, *NOS3* rs1799983, *HGF* rs1011694, *HGF* rs5745697, *MCT1* rs104943426, *ADAMTS14* rs4747096, *CASP8* rs3834129, *IL1A* rs1800587, *MLCK* rs2700352, *HGF* rs5745678, *VDR* ApaI, and *GEFT* rs11613457, reviewed by Lim et al. [50], may also influence muscle injury risk but were not assessed in this study. While our findings provide preliminary evidence of higher injury odds in XX carriers, it is important to emphasize that these observations are specific to the female volleyball cohort studied. Biological differences between males and females, including hormonal and muscle composition factors, may modulate the effects of the *ACTN3* R577X polymorphism. On average, females possess approximately 10% fewer fast-twitch muscle fibers [51] and about tenfold lower circulating testosterone levels compared with males [52]. These differences may influence muscle contractile properties, reparative capacity, and susceptibility to non-contact injuries. For example, α-actinin-3 deficiency in XX carriers may interact with lower testosterone and reduced fast-twitch fiber proportion in females to increase injury risk, whereas males may exhibit distinct responses due to higher androgen levels and a greater proportion of type II fibers. Therefore, our injury-related conclusions should not be extrapolated to male athletes without further investigation. Sparse cells (only one non-injured XX) required exact tests; accordingly, we used two-sided Fisher’s exact tests and penalized (Firth) logistic regression. Wide confidence intervals and increases in model LR *p*-values with covariate adjustment indicate limited statistical power and a non-negligible risk of false-positive inference. Data on years of experience and training load were unavailable, so residual confounding cannot be excluded and is acknowledged as a major limitation.

In conclusion, the *ACTN3* X allele was under-represented in professional volleyball players compared with controls in our pooled analysis. Within our team cohort, there was borderline/exploratory evidence of higher injury odds in XX carriers based on exact and penalized small-sample methods, although estimates were imprecise. These observations are hypothesis-generating and not intended for clinical screening. However, by linking genotype information to injury susceptibility, our findings highlight the potential of genetic analysis to guide future work in personalized training and injury prevention strategies. Replication and meta-analyses are required, as prior studies in sports genetics have demonstrated both consistent and inconsistent associations [53,54,55,56,57]. Further studies across diverse populations, sports disciplines, and injury types are needed to clarify *ACTN3*′s predictive value and its potential interactions with hormonal, environmental, and training-related factors.

## Figures and Tables

**Table 1:** Characteristics of the 50 Turkish female volleyball players involved in the muscle injury study.

Variables	Values	
Age		
Mean (SD)	26.2 (4.65)	
Median (min–max)	25.5 (19–38)	
Height (cm)		
Mean (SD)	178.9 (8.54)	
Median (min–max)	180.0 (158–199)	
Body mass (kg)		
Mean (SD)	67.9 (7.6)	
Median (min–max)	68.5 (50–83)	
Position	*n*	%
Libero	12	24.0
Middle player	15	30.0
Setter	9	18.0
Spiker	14	28.0

**Table 2:** Distribution of *ACTN3* genotypes and allelic frequencies in athletes and controls.

Group	*n*	*ACTN3* Genotypes			Alleles, %		*p* Value
		RR	RX	XX	R Allele	X Allele	
Turkish volleyball players	50	19	21	10	59.0	41.0	0.2752
Turkish controls	557	NA	NA	127	53.3	46.7	
Russian volleyball players	137	61	62	14	67.2	32.8	0.0285 *
Russian controls	4638	NA	NA	716	60.6	39.4	

* *p* < 0.05, indicating statistically significant differences in allelic frequencies between athletes and ethnicity-matched controls. Control data were obtained from the Turkish Genome Project [22] and the RUSeq database [25].

**Table 3:** Association between *ACTN3* genotypes and the incidence of muscle injuries in Turkish volleyball players.

Groups	*n*	*ACTN3* Genotypes					
		RR		RX		XX	
		*n*	%	*n*	%	*n*	%
Injured athletes	30	11	57.9	10	47.6	9	90.0 *
Non-injured athletes	20	8	42.1	11	52.4	1	10.0

* The odds of injury were higher in XX carriers (exact OR = 7.87, 95% exact CI = 0.94–374.58; *p* = 0.0366). For the overall 3 × 2 table, the two-sided Fisher’s exact test was borderline (*p* = 0.0617).

**Table 4:** Comparative analysis of genotype, playing position, and muscle injury status in relation to age, height, and body mass in Turkish female volleyball players.

Variables	*n*	Age Median (Min–Max)	Height Median (Min–Max)	Body Mass Median (Min–Max)
Genotype				
RR	19	26.0 (19–38)	180.0 (158–188)	69.0 (50–83)
RX	21	25.0 (19–29)	180.0 (161–199)	68.0 (50–83)
XX	10	26.0 (21–37)	181.0 (170–196)	69.0 (63–83)
*p*		0.691 ^a^	0.834 ^a^	0.957 ^a^
Post hoc		-	-	-
Position				
Libero	12	22.5 (20–38)	168.0 (158–179)	62.0 (50–69)
Middle player	15	27.0 (19–37)	185.0 (173–196)	70.0 (61–83)
Setter	9	26.0 (21–36)	180.0 (175–183)	65.0 (59–76)
Spiker	14	27.0 (19–34)	182.0 (174–199)	72.0 (60–83)
*p*		0.460 ^a^	<0.001 ^a^	<0.001 ^a^
Post hoc		-	1–3, 1–4	1–2, 1–4
Muscle injuries				
Yes	30	24.0 (19–38)	175.0 (158–199)	66.0 (50–83)
No	20	27.5 (19–37)	181.5 (168–196)	69.0 (59–83)
*p*		0.099 ^b^	0.132 ^b^	0.190 ^b^

^a^: Kruskal–Wallis H test, ^b^: Mann–Whitney U test, *p* < 0.05 indicates statistical significance.

**Table 5:** Multivariable (Firth) logistic regression for injury (outcome), genotype coded XX vs. RR + RX.

Model	Adjustments	Or (XX vs. Non-XX)	95% Ci (Penalized)	Model Lr *p*
Unadjusted		5.74	1.16–57.09	0.031
Adjusted 1 (primary)	Age + Playing position	5.92	1.12–60.98	0.092
Adjusted 2 (sensitivity)	Age + Position + Height + Body mass	5.22	1.02–52.00	0.314

Note: Penalized (Firth) logistic regression; CIs are profile-likelihood. “Model Lr *p*” refers to the likelihood-ratio *p*-value for the full model vs. null. Covariate coefficients are available upon request. Due to the small sample size, the genotype effect is reported as the primary parameter of interest.

## Data Availability

All data supporting the findings of this study are available within the paper.

## Abbreviations

The following abbreviations are used in this manuscript:

ACTN3	α-actinin-3
DNA	Deoxyribonucleic acid
PCR	Polymerase chain reaction
SNP	Single-nucleotide polymorphism
